# Evaluation and Observation of Autogenous Healing Ability of Bond Cracks along Rebar

**DOI:** 10.3390/ma7043136

**Published:** 2014-04-17

**Authors:** Choonghyun Kang, Minoru Kunieda

**Affiliations:** 1 School of Architecture & Civil Engineering, Kyungpook National University, 80, Daehak-ro Buk-gu, Daegu 702-701, Korea; 2 Department of Civil Engineering, Faculty of Engineering, Gifu University, 1-1 Yanagido, Gifu 501-1193, Japan; E-Mail: kunieda@gifu-u.ac.jp

**Keywords:** autogenous healing, bond cracks, air permeability test, crack pattern, ink injection

## Abstract

Micro cracks occurring in concrete around tensile rebar is well known latent damage phenomenon. These micro cracks develop, and can be detected after reaching the surface of the concrete. Detection of these cracks before they are fully formed is preferable, but observing the whole crack structure is difficult. Another problem is repairing micro cracks under the concrete surface. The autogenous ability of bond cracks along rebar was evaluated using the air permeability test. Air permeability coefficients were measured before and after tensile loading, and experimental air permeability coefficients became larger near cracks along rebar as a result of tensile loading. Recuring for 28 days after tensile loading made the air permeability coefficients smaller, but this restriction only occurred during water recuring. Observation of crack patterns helped the understanding of change in the air permeability coefficients. Several small cracks along rebar were observed after tensile loading, and most cracks along rebar were not found after water recuring. On the other hand, the crack pattern did not change after air recuring. These results indicate that bond cracks along rebar can be closed by autogenous healing, and cause the air permeability coefficients.

## 1. Introduction

Bond cracks are a form of damage that can occur in concrete around rebar, which were reported by Goto [1]. Several mechanisms of bond behavior have been investigated, such as chemical bonding, frictional bonding, mechanical bonding as a result of ribs, *etc.* Micro cracks were induced by these bonds between rebar and concrete, and it develops itself by several reasons such as relation with aggregate, crack bridging or post peak reaction [2–4]. The bond cracks may accelerate the penetration of substances such as water and gas, and it also makes the rebar corrosion area wider. These micro cracks should be repaired before significant damage develops. However, there is no way to detect these micro cracks under a thick concrete cover. In addition, the repair method has not been determined.

Autogenous healing of cementitious composite was well known phenomenon of automatic crack recovery. Focus was placed on this phenomenon as a repair method, but the applicability of autogenous healing is limited because several required circumstances must be satisfied for autogenous healing to occur. These circumstances include the mix proportion of cementitious materials, presence of water, crack width, water pressure, and stability of the crack, *etc.* [5–7]. Most past researches have concerned autogenous healing ability of bending cracks. As mentioned above, recovery of bond cracks along rebar is one of the applications, because these cracks are very fine and difficult to repair by ordinary repair techniques such as a crack injection.

In this study, autogenous healing was investigated as a recovery mechanism for bond cracks along rebar. Autogenous healing is a viable method because cracks along rebar are widely distribute, but small. The method of detection was the air permeability test, which is a non-destructive method using surface measurements. The measured air permeability coefficient was used to measure the degree of damage caused by the bond cracks, and to evaluate the effect of autogenous healing. In addition, observation of crack patterns using ink injection visually showed the amount of area recovered by the autogenous healing process.

## 2. Experimental Investigation

### 2.1. Specimen

**Figure 1.**
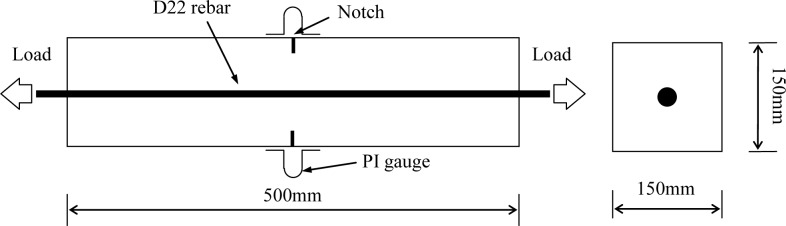
Shape of specimen.

Figure 1 shows the shape of the concrete specimen. Rectangle specimens with dimensions of 150 mm × 150 mm × 500 mm were prepared. The size of the cross sections was considered in measuring the air permeability. D22 rebar was embedded in the center of each specimen to induce tensile force. Notches having a depth of 20 mm and a thickness of 3 mm were made at both sides and center of the specimen.

Normal concrete and fly ash concrete were used in this study. Termkhajornkit *et al*., reported that C-S-H gel produced by the pozzolanic reaction of fly ash acted to seal micro cracks [8,9] The mix proportions are in Table 1. The water to cement ratio (W/C) was 45% in all mixes. Ordinary Portland cement with a density of 3.15 g·cm^−3^, sand with a surface-dry density of 2.51 g·cm^−3^, a coarse aggregate with a surface-dry density of 2.58 g·cm^−3^ and with a maximum size of 20 mm were used. Fly ash (Type II of JIS A 6210, Techno Chubu Company, Nagoya, Japan), which has a density of 2.38 g·cm^−3^, was also used.

### 2.2. Tensile Loading

**Figure 2.**
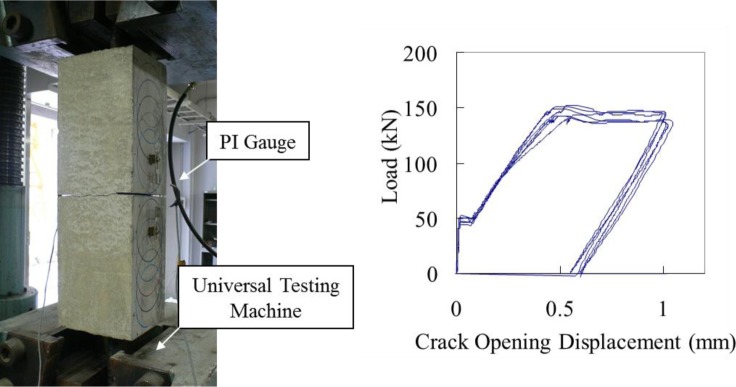
Experimental setup and load-displacement relationship.

Tensile loading was performed to induce bond cracks along rebar. The tensile force was applied by the rebar embedded in the center of the specimen. Crack opening displacement, which was measured by two PI gauges attached to the specimen surface over the notches, was monitored during the loading tests. Figure 2 shows the experimental setup and load-crack opening displacement relationship. Several pretests were performed to determine the unloading level (0.5, 1.0 and 2.0 mm). Consequently, an unloading point of 1.0 mm was applied in this study.

### 2.3. Air Permeability Test

**Figure 3.**
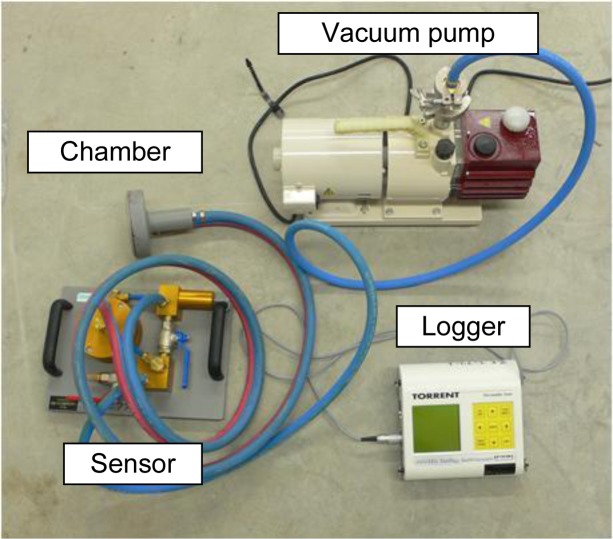
Torrent Permeability Tester.

**Figure 4.**
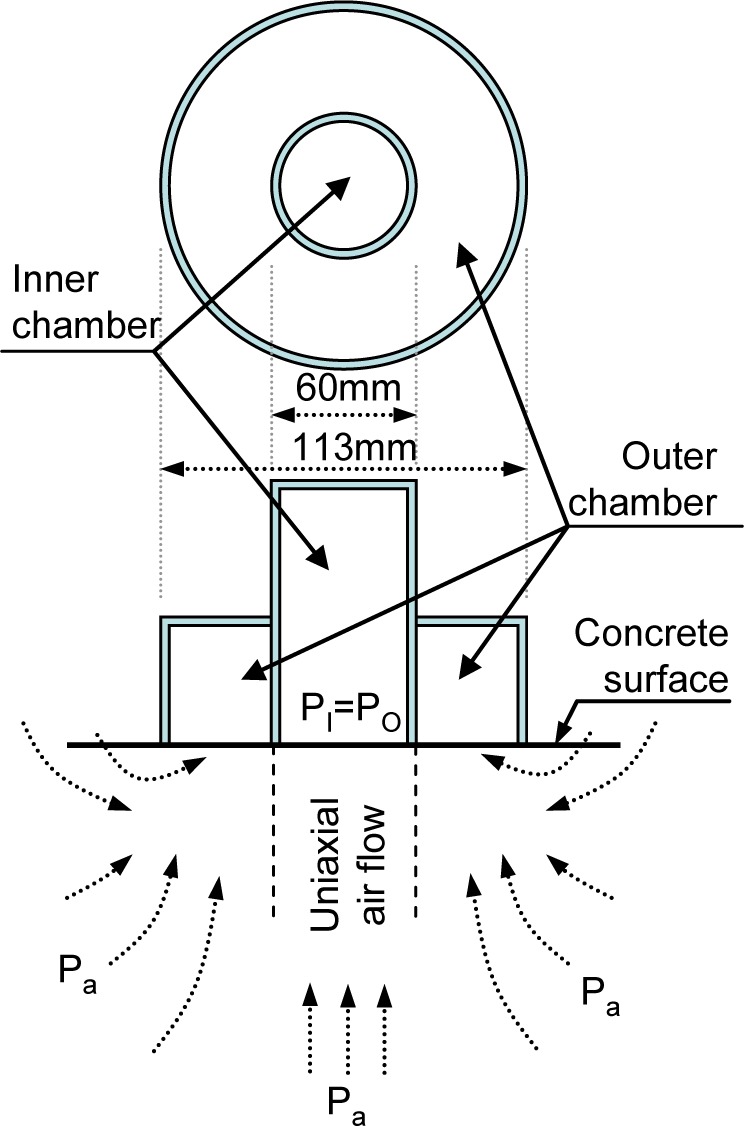
Conceptual diagram of the chamber and air flow.

A sensitive index needed to be selected to evaluated the degree of damage due to bond cracks and the recovery effect of the autogenous healing phenomenon. Air permeability tests were conducted using the Torrent Permeability Test (TPT), proposed by Torrent [10]. As shown in Figure 3, the device consisted of chamber, vacuum pump, pressure sensor, and logger. To prevent spurious ingress of air along the skin, there are two chambers: an outer chamber and an inner chamber. After the desired level of vacuum was attained, the pump only acted on the outer chamber. Meanwhile, the logger recorded the history of change in the inner chamber. A spurious ingress from the concrete surface is evacuated by outer chamber, and uniaxial air flow can be measured through the pressure of both chambers, as shown in Figure 4. After twelve minutes of data logging, the air permeability coefficient was calculated using Equation (1) based on the theoretical model:

$$k=4{\left(\frac{V_{c}(dP_{1}/dt)}{A(P_{a}^{2}-P_{I}^{2})}\right)}^{2}\frac{\mu P_{a}}{\varepsilon }\int_{t_{0}}^{t}[1-{\left(\frac{P_{I}}{P_{a}}\right)}^{2}]dt$$ (1)

Here *k* is the air permeability coefficient (m^2^), *V*_c_ is the volume of the inner chamber (m^3^), *A* is the cross-sectional area of the inner chamber (m^2^), *P*_1_ is the pressure in inner chamber (N/m^2^), *P*_a_ is the atmospheric pressure (N/m^2^), μ is the dynamic viscosity of air (Ns/m^2^), and ε is the empty porosity of the concrete.

**Figure 5.**
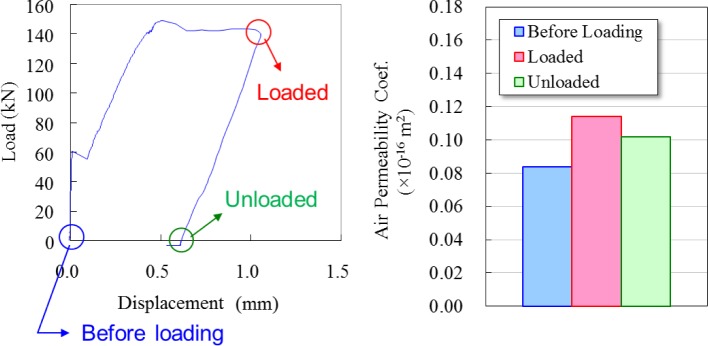
Difference in the air permeability coefficient between loading and unloaded condition.

The air permeability coefficient can be directly influenced by the vertical crack due to bond cracking along rebar. In addition, this study suggests that losing concrete matrix between the surface and rebar because of tensile loading can also influence the air permeability coefficient. The air permeability coefficient had different values depending on the timing of the measurement, such as during loaded and unloaded conditions. As expected, the air permeability coefficient during loading was higher than that of the unloaded condition, there being about a 10% difference, as shown in Figure 5. Actually, the air permeability coefficient during loading was more appropriate. Measuring the air permeability was done during unloaded conditions as well.

**Figure 6.**
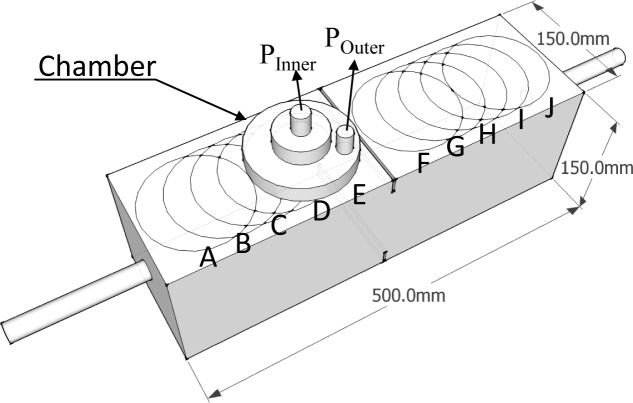
Measurement points in the air permeability test.

Ten measurement points (A to J) were selected along the rebar, as shown in Figure 6. Measurements were taken three times: before and just after loading and after recuring (autogenous healing). Each air permeability coefficient was compared to evaluate the damage and recovery effect.

### 2.4. Crack Pattern Observation

**Figure 7.**
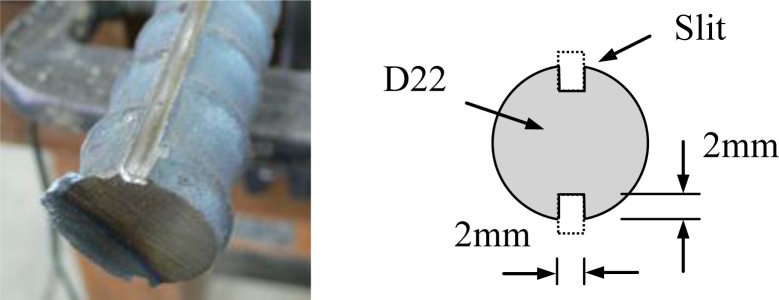
Slits in rebar for ink injection.

The crack pattern was observed to evaluate the healing effect. The specimen size used was the same as the specimen shown in Figure 6, but the rebar was modified. Slits for ink injection were made on both sides of the rebar, as shown in Figure 7, based on previous research [11]. Slits were cut by a grinder, and were filled with styrofoam to prevent them from being filled by cement paste. Styrofoam was removed before ink injection. Red ink was injected into the slits, and the specimen was split in half using a concrete cutter.

During tensile loading, the peak loads of specimens containing rebar with slits were slightly lower than the normal specimen in air permeability test, and the residual displacement was also different. However, the difference in loads was about 5% and the unloading point was almost the same.

### 2.5. Procedures

**Figure 8.**
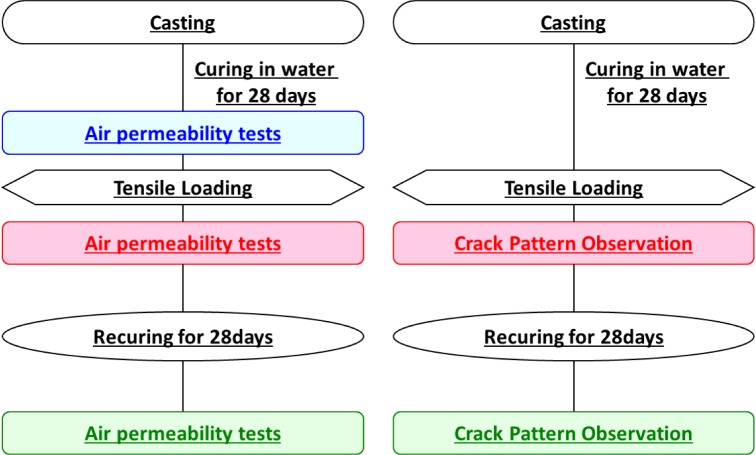
Experimental procedures.

All specimens were demoulded after 24 h after the concrete was cast, and cured in 20 °C water. Three specimens were prepared for the air permeability test in each series. Tensile loading was performed after twenty-eight days. Before and after the tensile loading, the air permeability coefficient was measured after 3–5 h natural drying to prevent the effects of moisture. Different recuring conditions were applied to each series after tensile loading. Air recuring refers to specimens recurred in a 20 °C curing room with are lative humidity(RH) of 70%–80%, and one specimen was tested under this condition. On the other hand, water recuring refers to specimens recurred in 20 °C water located in a curing room. The remaining two specimens were tested in these conditions. Both recuring methods remained stable. By comparing the air permeability coefficient after recuring in air and water, more appropriate circumstances for autogenous healing could be investigated. Another three specimens were prepared for crack pattern observation. Crack patterns were first observed just after the tensile loading. The final two specimens recured after the tensile loading in air and water respectively. After recuring, the effect of autogenous healing was investigated through crack pattern observation. Figure 8 shows the outline of the experimental procedures.

## 3. Experimental Result

### 3.1. Results of the Air Permeability Test

**Figure 9.**
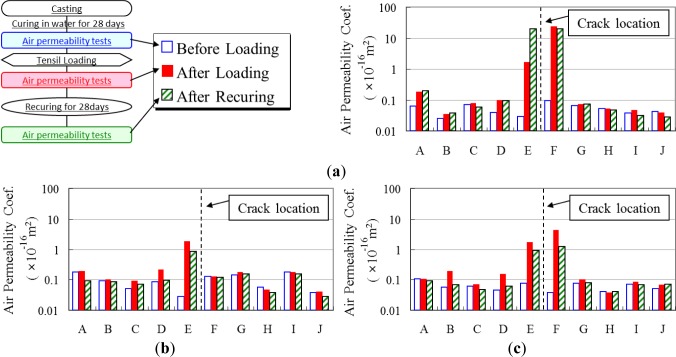
Normal concrete air permeability test results. (**a**) Recuring in air; (**b**) Recuring in water—Specimen No.1; (**c**) Recuring in water—Specimen No.2.

Figure 9 shows the air permeability coefficients of each normal concrete specimen before the tensile loading, after tensile loading, and after recuring. Air permeability coefficients of all specimens before the tensile loading were extremely similar near 0.1 × 10^−16^ m^2^. After the tensile loading, air permeability coefficients became larger, and a relatively significant change was observed at D–G. This seemed to be influenced by the main crack caused by notches. After recuring, air permeability coefficients showed different aspects depending on the recuring condition. The air permeability coefficient of the specimen recured in water became smaller than that just after loading. It seems that recovery of micro cracks along rebar occurred during water recuring. However, there was no recovery trend in the specimen recured in air.

**Figure 10.**
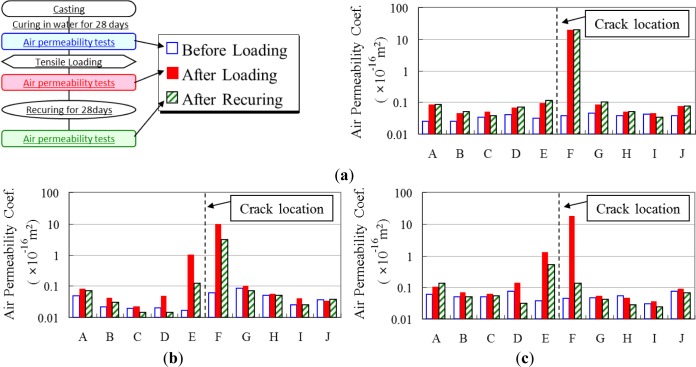
Fly ash concrete air permeability test results. (**a**) Recuring in air; (**b**) Recuring in water—Spceimen No.1; (**c**) Recuring in water—Specimen No.2.

Figure 10 shows the results of the fly ash concrete specimen. The average air permeability coefficient was a little smaller than the normal concrete specimens. However, both specimens were generally the same, and an insignificant difference was observed.

### 3.2. Results of Crack Pattern Observation

**Figure 11.**
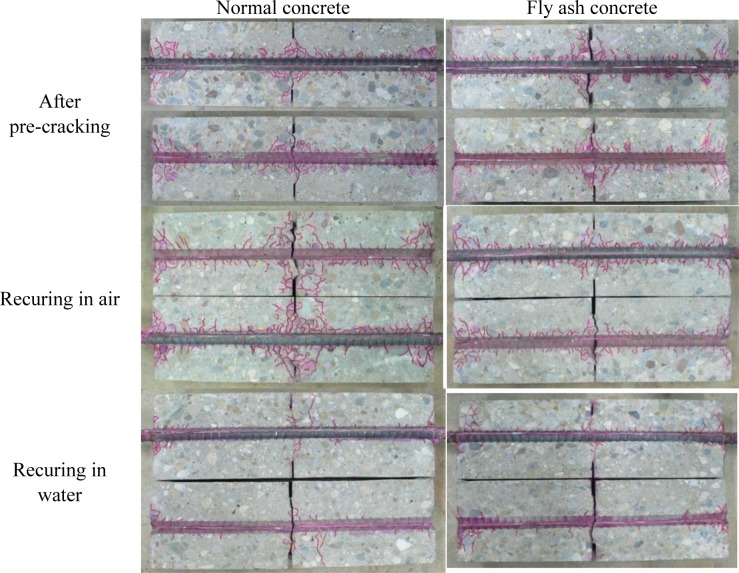
Observed crack patterns.

The recuring effect was visually elucidated by crack pattern observation. Figure 11 shows the dyed crack pattern of each specimen. After tensile loading, micro bond cracks less than 2 mm long were observed near the rebar. Regardless of concrete type, a few cracks were observed after water recuring. It seems that generated products due to autogenous healing filled the micro cracks, and also influenced the air permeability coefficient. However, the observed crack pattern in specimens recured in air was similar to that just after the tensile loading. This means that autogenous healing during air recuring was not as significant as healing during water recuring.

Although air permeability coefficients showed lower values in fly ash concrete than normal concrete, it is difficult to find a significant difference in the crack patterns between these two types. It seems that the air permeability coefficient is more sensitive than can be detected by observation using the naked eye.

## 4. Conclusions

In this study, the autogenous healing ability of bond cracks along rebar was experimentally evaluated using the air permeability coefficient and crack pattern observation. The following conclusions are made:

(1) The air permeability coefficient measured at the surface of a specimen was influenced by the bond crack along the rebar by tensile loading. The air permeability coefficient became larger as a result of damage done. Note that macro cracks were induced at the notched part, and micro cracks were not observed on the surface. The air permeability coefficient, however, became smaller because of water recuring. It seems that the rehydration products of cement filled the micro bond cracks. In this study the recuring period was twenty-eight days, and more significant recovery can be assumed with longer recuring period.
(2) Bond crack recovery was investigated by observing crack patterns just after tensile loading, as well as after recuring. A little crack was observed in the specimens that underwent water recuring. On the other hand, crack patterns of the specimen which underwent air recuring were almost the same as those just after tensile loading. The results of the air permeability test indicated that recuring in water was more conducive to autogenous healing.
(3) The adopted curing condition such as water curing might be difficult to apply to existing structures, and further research includes moisture conditions and its criteria should be investigated.

## Figures and Tables

**Table 1.** Mix proportions.

Case	W/C (%)	s/a (%)	Unit Content (kg·m^−3^)					Admixture (cc·m^−3^)

			Water	Cement	Sand	Gravel	Fly Ash	AEA ^†^
Normal Concrete	45	47	170	377	780	903	–	0.97
Fly ash Concrete	45	47	170	377	716	903	58	0.97

AEA ^†^: Air entraining agent.
